# miRNA Alterations Elicit Pathways Involved in Memory Decline and Synaptic Function in the Hippocampus of Aged Tg4-42 Mice

**DOI:** 10.3389/fnins.2020.580524

**Published:** 2020-09-10

**Authors:** Yvonne Bouter, Tim Kacprowski, Fanny Rößler, Lars R. Jensen, Andreas W. Kuss, Thomas A. Bayer

**Affiliations:** ^1^Division of Molecular Psychiatry, Department of Psychiatry and Psychotherapy, University Medical Center Göttingen (UMG), Georg-August-University, Göttingen, Germany; ^2^Research Group Computational Systems Medicine, Chair of Experimental Bioinformatics, TUM School of Life Sciences Weihenstephan (WZW), Technical University of Munich (TUM), Weihenstephan, Germany; ^3^Human Molecular Genetics Group, Department of Functional Genomics, Interfaculty Institute for Genetics and Functional Genomics, University Medicine Greifswald, Greifswald, Germany

**Keywords:** miRNA transcriptome, miRNA-Seq, NGS, transgenic mouse model, Tg4-42, Alzheimer

## Abstract

The transcriptome of non-coding RNA (ncRNA) species is increasingly focused in Alzheimer’s disease (AD) research. NcRNAs comprise, among others, transfer RNAs, long non-coding RNAs and microRNAs (miRs), each with their own specific biological function. We used smallRNASeq to assess miR expression in the hippocampus of young (3 month old) and aged (8 month old) Tg4-42 mice, a model system for sporadic AD, as well as age-matched wildtype controls. Tg4-42 mice express N-truncated Aβ_4–42_, develop age-related neuron loss, reduced neurogenesis and behavioral deficits. Our results do not only confirm known miR-AD associations in Tg4-42 mice, but more importantly pinpoint 22 additional miRs associated to the disease. Twenty-five miRs were differentially expressed in both aged Tg4-42 and aged wildtype mice while eight miRs were differentially expressed only in aged wildtype mice, and 33 only in aged Tg4-42 mice. No significant alteration in the miRNome was detected in young mice, which indicates that the changes observed in aged mice are down-stream effects of Aβ-induced pathology in the Tg4-42 mouse model for AD. Targets of those miRs were predicted using miRWalk. For miRs that were differentially expressed only in the Tg4-42 model, 128 targets could be identified, whereas 18 genes were targeted by miRs only differentially expressed in wildtype mice and 85 genes were targeted by miRs differentially expressed in both mouse models. Genes targeted by differentially expressed miRs in the Tg4-42 model were enriched for negative regulation of long-term synaptic potentiation, learning or memory, regulation of *trans-*synaptic signaling and modulation of chemical synaptic transmission obtained. This untargeted miR sequencing approach supports previous reports on the Tg4-42 mice as a valuable model for AD. Furthermore, it revealed miRs involved in AD, which can serve as biomarkers or therapeutic targets.

## Introduction

MiRs are short non-coding RNAs that are heavily involved in post-transcriptional regulation of gene expression through targeting specific mRNAs (Bartel, 2004). The miR transcriptome (miRNome) includes all miR species and renders a profile of gene regulation at the studied time point and location. Altered miR expression profiles therefore provide information not only about the miRs themselves, but also about their target genes and, hence, the regulated processes. As miRs have already been implicated in neurodevelopment and brain aging, as well as in synapse function and cognitive performance, in both, health and disease (Salta and De Strooper, 2012, 2017b), this information can pinpoint mechanisms involved in the molecular pathogenesis of Alzheimer’s disease (AD). Furthermore, miRs can easily be targeted by artificial oligonucleotides to influence gene regulation for therapeutic processes (Roshan et al., 2009). Hence, miRNome profiling ultimately promotes the search for new biomarkers and therapeutics.

Next-generation sequencing (NGS) of the miR pool offers an unbiased technical approach to identify the miR signature of cells, tissues or organs. Previous reports have demonstrated the power of NGS to unravel the molecular profile of pathological alterations in neurodegenerative diseases like AD (Twine et al., 2011). Differentially expressed genes (DEGs) were identified by RNASeq of young, and aged Tg4-42 mice in comparison with 5XFAD mice, another AD mouse model (Bouter et al., 2014). Many of the DEGs specifically found in the 5XFAD model belong to neuroinflammatory processes typically associated with plaques. Other DEGs were found in both AD mouse models indicating common disease pathways associated with behavioral deficits and neuron loss. The 5XFAD model develops early plaque formation, intraneuronal Aβ aggregation, neuron loss, and behavioral deficits (Oakley et al., 2006; Jawhar et al., 2012). As such the 5XFAD model is widely used in the AD field. High-throughput RNASeq analysis of young 5XFAD mice, e.g., identified DEGs in the frontal cortex mainly associated with cardiovascular disease and DEGs in the cerebellum mainly associated with mitochondrial dysfunction (Kim et al., 2012).

A plethora of previous publications discuss miRs as potentially involved in the pathogenesis of AD and/or as putative biomarkers for AD including several systematic assessments of the importance of miRs in this disorder [reviewed for example by Salta and De Strooper (2012, 2017b), Angelucci et al. (2019), Wang et al. (2019)]. Notably, the expression of miR-338-5p was significantly down-regulated in the hippocampus of patients with AD and 5XFAD transgenic mice (Qian et al., 2019). The expression of miR-146a correlated with plaque load and synaptic pathology in Tg2576 and in 5XFAD mice (Li et al., 2011).

We have performed NGS of the miRNome of the hippocampus of young (3 month old) and aged (8 month old) Tg4-42 mice, which represent a unique model for sporadic AD. The Tg4-42 model expresses only wildtype non-mutant Aβ4–42 and develops at the age of 8 months, severe neuron loss and hippocampus-related behavioral deficits (Bouter et al., 2013). At 3 months of age, Tg4-42 mice show reduced neurogenesis (Gerberding et al., 2019) and synaptic hyperexcitability (Dietrich et al., 2018), which represents an early sign of AD-typical alteration. Reduced glucose metabolism detected by FDG-PET *in vivo* imaging (Bouter et al., 2019) correlates well with the observed neuron loss and neurological deficits at 8 months of age (Bouter et al., 2013). The aim of the current study was to identify miRs and their targets to unravel molecules and processes triggered by AD-typical memory deficits and hippocampal neuron death. Therefore, we compared the age of 3 months (prior to neuron loss and neurological alterations) with the age of 8 months (after onset of neuron loss and neurological alterations).

## Materials and Methods

### Transgenic Mice

We used the transgenic mouse lines Tg4-42 kept on the C57Bl/6J genetic background. Tg4-42 mice express human Aβ_4–42_ fused to the murine TRH signal peptide under the control of the neuronal Thy-1 promoter (Bouter et al., 2013). Young (3 month) and aged (8 month) Tg4-42 and age-matched wildtype control mice (WT, C57BL/6J) were studied. All animals were handled according to the German guidelines for animal care. All efforts were made to minimize suffering and the number of animals used for this study.

### Tissue Harvesting

Mice were sacrificed via CO_2_ anaesthetization followed by cervical dislocation. Brain hemispheres were carefully dissected and the hippocampus removed, frozen on dry ice and stored at −80°C for subsequent use.

### Small RNA Next-Generation Sequencing

Small RNA-isolation was performed using the miRVana miRNA Isolation Kit (Life Technologies) according to the manufacturer’s instructions. For library preparation, we used the Ion Total RNA-Seq Kit v2 for Small RNA Libraries (Life Technologies) for sequencing on an Ion PGM system (Thermo Fisher Scientific) running Torrent Suite software 5.12.1., again following the instructions of the manufacturer. STAR v2.6.0a with default parameters was used to map the reads to the GRCm38 (mm10) mouse assembly (Dobin et al., 2013). Afterward, read counts per feature were determined with HTSeq v0.10.0 (Anders et al., 2015). Bam files were submitted to the European Nucleotide Archive^1^ with the accession identification number of the project PRJEB39314.

### Differential Expression Analysis

During quality control of the mapped data, RNAs with less than 10 readcounts across all samples were excluded and technical replicates were collapsed after heatmap inspection (Supplementary Figure S1). Transcripts with less than 10 reads across all remaining samples were discarded. The heatmap in Supplementary Figure S1 was compiled with variance-stabilized data, while the differential expression analyses were conducted on the raw count data, due to DESeq2’s statistical model (Love et al., 2014). The comparisons were done across contrasts corresponding to the age groups or the genotypes, respectively. Correction for multiple testing was done via independent hypothesis weighting as implemented in DESeq2 (Love et al., 2014), and a result was deemed significant if FDR <0.05.”

### MiR Target and Overrepresentation Analysis

For the target mining of differentially expressed miRs (FDR<0.05) we used miRWalk version 3 (Sticht et al., 2018), which uses a random forest based algorithm, and TarPmiR (Ding et al., 2016), to predict possible miR targets. It also allows to compare the results with the predictions of other target mining algorithms such as TargetScan (Agarwal et al., 2015) and miRDB (Chen and Wang, 2020), as well as validated interactions from miRTarBase (Chou et al., 2018). Only gene targets that were confirmed by at least two of these databases were considered for the further analysis to minimize false positives. The analysis required unique miR names mapped to the specific strand. Hence, we used both miR-3p and miR-5p, if available. The identified targets were then used to conduct a Gene Ontology (GO) (Rigden and Fernandez, 2018) overrepresentation analysis with the PANTHER algorithm (Mi et al., 2019) relying on the GO release of December 9th, 2019, to check for biological processes that are targeted by the differentially expressed miRs.

## Results

### NGS of Non-coding RNAs in Mouse Hippocampus

Small RNASeq yielded 761,373 and 1,054,109 raw sequence reads for young and old wildtype (WT) mouse hippocampi, respectively. For young WT mice on average 160,723 reads (*n* = 4; on average 84% of raw reads) and for old WT mice 213,338 reads (*n* = 4; on average 81% of raw reads) per animal were mapped.

For Tg4-42 mice a total of 758,968 (4 young animals) and 2,252,799 (4 old animals, each sequenced twice) raw reads were produced. In young Tg4-42 mice, mapping was successful for on average 160,808 reads per sample (on average 85% of raw reads) and for old Tg4-42 mice on average 449,797 mapped reads per animal (224,899 reads per sample; on average 80% of raw reads) were obtained.

### Analyses of miRs at 3 and 8 Months of Age in Tg4-42 and Wildtype Mice

**FIGURE 1:**
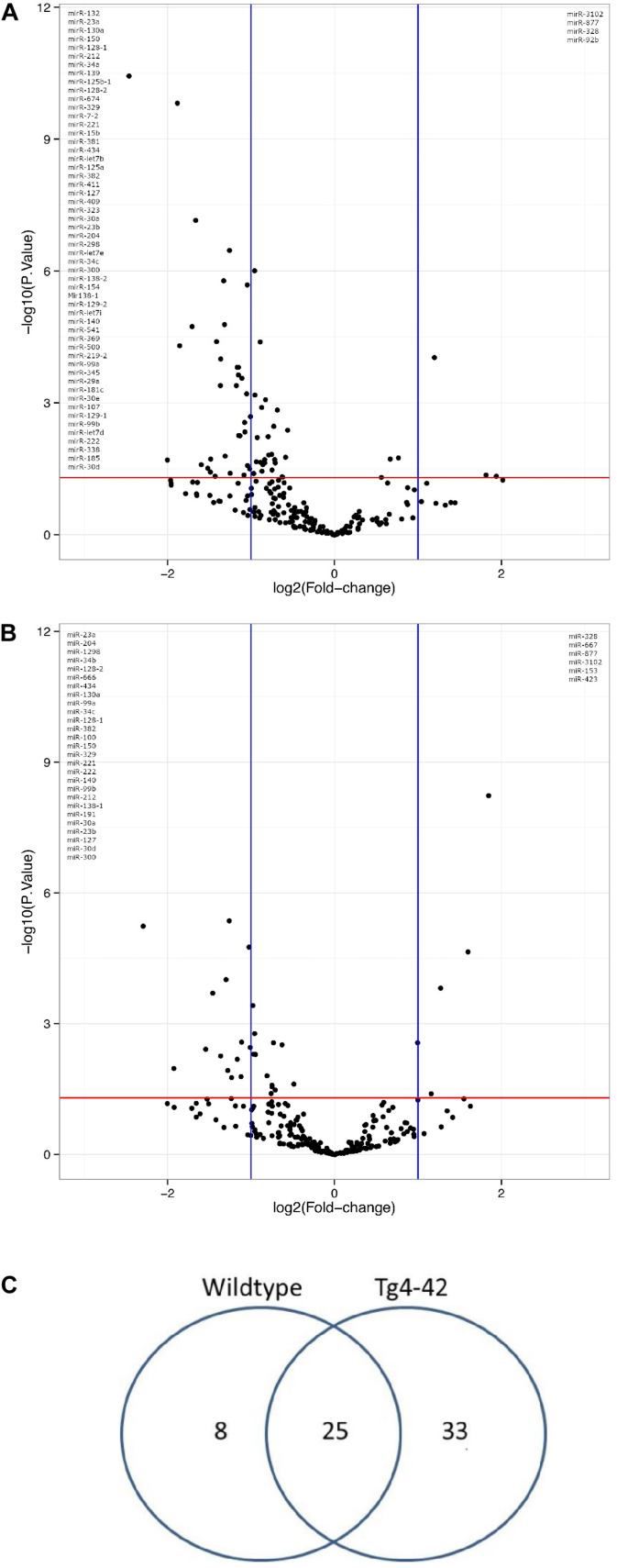
Genotyp-specific expression of miRs with significantly altered expression level during aging. Volcano plot comparison of the fold changes and values of miR expression in WT **(A)** and Tg4-42 **(B)** mice. The vertical lines correspond to 2-fold up and down, respectively, and the horizontal line represents a *p*-value of 0.05. **(C)** miRs in hippocampus of young (3 month old) and aged (8 month old) Tg4-42 and WT mice. Total number of significantly altered miR expression levels between 3 and 8 months of age *n* = 58; 8 in WT, 33 in Tg4-42 and 25 in both WT and Tg4-42 mice.

**FIGURE 2:**
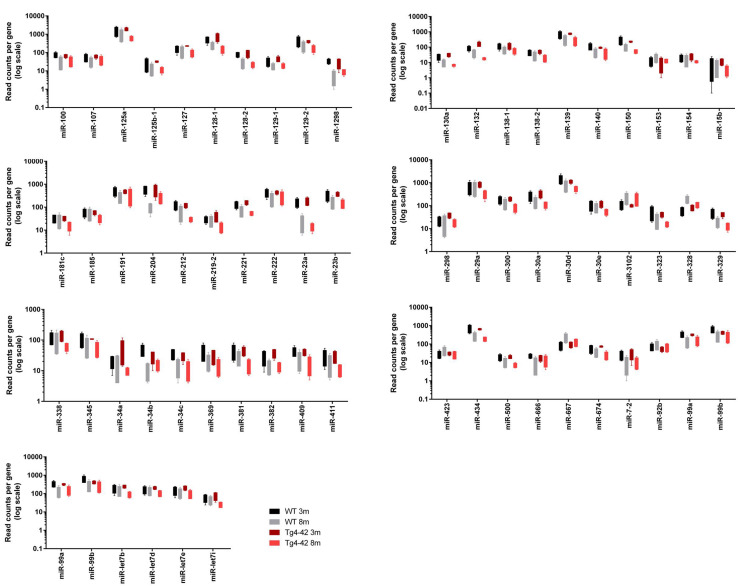
Box plots of significantly altered miRs in hippocampus of young (3 month old; 3m) and aged (8 month old; 8m) Tg4-42 and WT.

In order to demonstrate the expression and significant distribution of identified miRs volcano plots were created (Figures 1A,B). In total, 237 miRs were detected. There was a significant change in expression of 58 miRs observed between young and aged Tg4-42, as well as between young and aged WT mice (Figure 1C). No significant differences were observed across genotypes within an age group (data not shown). Of 33 differentially expressed miRs exclusively found in aged Tg4-42 mice, some were reported to be association with AD. The remaining miRs could now be associated with an AD-typical mouse model for the first time. Figure 2 demonstrates the levels of all miRs with a significantly altered expression level between 3 and 8 months of age either in Tg4-42, WT or in both. In aged Tg4-42 mice, mostly decreased levels of miRs have been observed.

**FIGURE 3:**
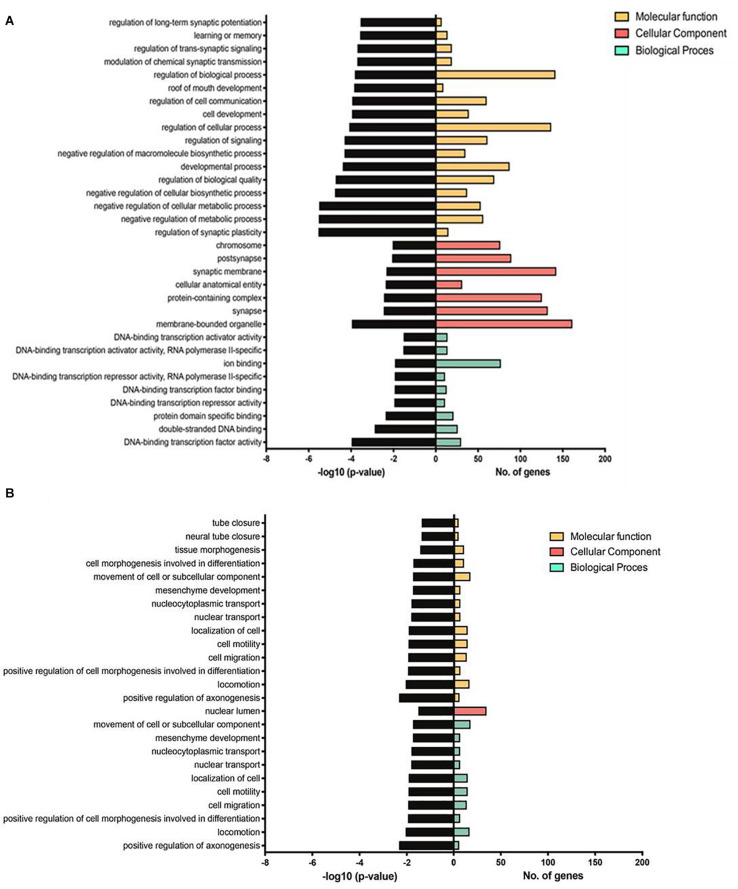
GO annotation analysis of predicted miR targets in Tg4-42 **(A)** and WT mice **(B)**. Number of genes enriched and –log10 (*P*-value) for each term are displayed for the top 17 GO terms, if applicable, in molecular function, cellular component and biological process.

Table 1 lists references for known links to AD, other neurodegenerative disorders and/or brain function. Five miRs identified in aged WT mouse brain were increased and four decreased during aging (Table 2). MiRs found in both Tg4-42 and WT hippocampi were decreased during aging, only one was increased (Table 3). The GO annotation analysis of predicted miR targets in Tg4-42 revealed that reduced long-term synaptic potentiation, learning or memory, regulation of *trans-*synaptic signaling and modulation of chemical synaptic transmission obtained top scores (Figure 3A). None of these annotations were overrepresented in WT mice (Figure 3B). The GO analysis of predicted miR targets in either Tg4-42 or WT mice or both elicited also other cellular components (Table 4), molecular functions (Table 5) and biological processes (Table 6).

**TABLE 1:** MiRs significantly altered in hippocampus between 3 and 8 month-old exclusively in Tg4-42 mice.

	Expression	References
miR-107	−0.7	Wang W.X. et al., 2011; Shu et al., 2018
miR-125a	−1.2	Ifrim et al., 2015
miR-125b-1	−1.4	Banzhaf-Strathmann et al., 2014; Lardenoije et al., 2015; Lu et al., 2017; Salta and De Strooper, 2017b; Elliott et al., 2018; Jin et al., 2018; Ottaviani et al., 2019
miR-129-1	−0.7	
miR-129-2	−0.9	Strickland et al., 2011
miR-132	−2.5	Majer et al., 2012; Freischmidt et al., 2013; Bicker et al., 2014; Essandoh et al., 2016; Tasaki et al., 2018
miR-138-2	−1.0	Boscher et al., 2019
miR-139	−1.4	Noh et al., 2014; Lardenoije et al., 2015; Salta and De Strooper, 2017b
miR-154	−0.9	
miR-15b	−1.2	
miR-181c	−0.8	Lardenoije et al., 2015; Villela et al., 2016
miR-185	−0.6	Forstner et al., 2013; Wen et al., 2018
miR-219-2	−0.8	
miR-298	−1.0	
miR-29a	−0.8	Bettens et al., 2009
miR-30e	−0.7	Margis et al., 2011; Lardenoije et al., 2015; Salta and De Strooper, 2017b
miR-323	−1.1	Che et al., 2019
miR-338	−0.6	Chun et al., 2017
miR-345	−0.8	Freiesleben et al., 2016; Wei et al., 2020
miR-34a	−1.5	Sun et al., 2018
miR-369	−0.9	Serpente et al., 2011
miR-381	−1.2	Li et al., 2020
miR-409	−1.1	Liu et al., 2019
miR-411	−1.1	
miR-500	−0.8	
miR-541	−0.9	Zhang et al., 2011
miR-674	−1.3	Lardenoije et al., 2015; Salta and De Strooper, 2017b
miR-7-2	−1.3	
miR-92b	+0.6	
miR-let7b	−1.2	Lehmann et al., 2012; Derkow et al., 2018
miR-let7d	−0.7	Singh et al., 2018
miR-let7e	−1.0	Derkow et al., 2018
miR-let7i	−0.9	

*References are given for known links to Alzheimer’s disease, other neurodegenerative disorders and/or brain function. Supplementary Table S1 shows the entire list of miRs level changes. Decreased expression level at 8 month of age, −; increased expression level at 8 month of age, +.*

**TABLE 2:** MiRs significantly altered in hippocampus between 3 and 8 month-old exclusively in wildtype mice.

	Expression	References
miR-100	−1.1	Lu et al., 2017; Elliott et al., 2018; Ottaviani et al., 2019
miR-1298	−1.9	
miR-153	+1.2	Gui et al., 2015
miR-191	−0.7	Smith et al., 2010
miR-34b	−1.5	
miR-423	+1.0	
miR-666	−1.4	
miR-667	+1.6	

*References are given for known links to Alzheimer’s disease, other neurodegenerative disorders and/or brain function. Supplementary Table S1 shows the entire list of miRs level changes. Decreased expression level at 8 month of age, −; increased expression level at 8 month of age, +.*

**TABLE 3:** MiRs significantly altered in hippocampus between 3 and 8 month-old wildtype mice and Tg4-42 mice.

	Expression In WT mice	Expression In Tg4-42 mice	References
miR-127	−1.1	−0.7	Essandoh et al., 2016
miR-128-1	−1.7	↓ 1.2	Tiribuzi et al., 2014
miR-128-2	−1.4	−1.5	
miR-130a	−1.9	−1.3	Zhao et al., 2014
miR-138-1	−0.9	−0.8	Lin et al., 2018
miR-140	−0.9	−1.0	
miR-150	−1.8	−1.0	Punga et al., 2015
miR-204	−1.0	−2.3	Cammaerts et al., 2015
miR-212	−1.7	−0.8	Wang W.X. et al., 2011
miR-221	−1.3	−1.0	Seeley et al., 2018
miR-222	−0.6	−1.0	Wang et al., 2015; Seeley et al., 2018
miR-23a	−1.9	−2.6	Fenoglio et al., 2016
miR-23b	−1.0	−0.7	
miR-300	−1.0	−0.5	Li et al., 2018
miR-30a	−1.1	−0.7	Sun et al., 2016
miR-30d	−0.6	−0.6	
miR-3102	+1.2	+1.3	
miR-328	+0.7	+1.8	
miR-329	−1.3	−1.0	Urdinguio et al., 2010
miR-34c	−1.0	−1.2	Haramati et al., 2011
miR-382	−1.1	−1.1	Song et al., 2017
miR-434	−1.2	−1.3	
miR-877	+0.8	+1.5	Zhao et al., 2020
miR-99a	−0.8	−1.3	Bras et al., 2018
miR-99b	−0.7	−0.9	Cao et al., 2017

*References are given for known links to Alzheimer’s disease, other neurodegenerative disorders and/or brain function. Supplementary Table S1 shows the entire list of miRs level changes. Decreased expression level at 8 month of age, −; increased expression level at 8 month of age, +.*

**TABLE 4:** GO annotation and pathway enrichment analysis of predicted miR targets on Cellular Components.

			Tg4-42			WT		
				
	*Gene ontology*	*Label*	*FDR*	*No. genes*	*No. miRs*	*FDR*	*No. genes*	*No. miRs*
***Significant in Tg4-42 and WT***								
1	GO:0000785	Chromatin	2.24E-02	16	13	3.17E-02	11	9
2	GO:0005622	Intracellular	1.08E-04	161	34	8.21E-03	82	18
3	GO:0043231	Intracellular membrane-bounded organelle	3.51E-03	125	31	2.96E-02	66	18
4	GO:0043229	Intracellular organelle	1.18E-02	137	33	3.75E-02	73	18
5	GO:0005634	Nucleus	8.52E-03	89	30	1.56E-02	52	17
6	GO:0043226	Organelle	4.61E-03	142	33	8.78E-03	76	18
***Significant in Tg4-42 only***								
1	GO:0110165	Cellular anatomical entity	1.11E-02	189	35	2.87E-01	93	19
2	GO:0005694	Chromosome	4.53E-02	23	15	2.32E-01	13	9
3	GO:0043227	Membrane-bounded organelle	3.42E-03	132	32	7.72E-02	68	19
4	GO:0098794	Postsynapse	2.73E-02	18	10	9.06E-01	8	7
5	GO:0032991	Protein-containing complex	9.02E-03	76	26	1.00E+0	34	12
6	GO:0045202	Synapse	4.25E-03	31	15	1.00E+0	12	7
7	GO:0097060	Synaptic membrane	2.43E-02	14	7	1.00E+0	5	4
***Significant in WT only***								
1	GO:0031981	Nuclear lumen	1.65E-01	54	24	3.20E-02	35	13

*Benjamini–Hochberg False Discovery Rate (FDR).*

**TABLE 5:** GO annotation and pathway enrichment analysis of predicted miR targets on Molecular Function.

			Tg4-42			WT		
				
	*Gene ontology*	*Label*	*FDR*	*No. genes*	*No. miRs*	*FDR*	*No. genes*	*No. miRs*
***Significant in Tg4-42 and WT***								
1	GO:0005488	Binding	9.51E-05	159	36	1.22E-02	79	20
2	GO:0035326	*cis-*regulatory region binding	1.46E-03	20	15	2.17E-02	12	10
3	GO:0000987	*cis-*regulatory region sequence-specific DNA binding	1.35E-03	20	15	2.16E-02	12	10
4	GO:0003677	DNA binding	2.45E-04	42	21	1.69E-02	24	14
5	GO:0000981	DNA-binding transcription factor activity, RNA polymerase II-specific	2.35E-04	25	17	1.56E-02	14	11
6	GO:1901363	Heterocyclic compound binding	1.10E-03	75	28	2.48E-02	39	16
7	GO:0003676	Nucleic acid binding	1.08E-03	53	24	2.41E-02	29	16
8	GO:0097159	organic cyclic compound binding	1.62E-03	75	28	3.45E-02	39	16
9	GO:0005515	Protein binding	4.25E-06	128	33	3.60E-04	67	16
10	GO:0001067	regulatory region nucleic acid binding	5.16E-04	26	19	1.45E-02	15	12
11	GO:0000978	RNA polymerase II *cis-*regulatory region sequence-specific DNA binding	1.15E-03	20	15	2.09E-02	12	10
12	GO:0001012	RNA polymerase II regulatory region DNA binding	4.73E-04	25	18	2.00E-02	14	11
13	GO:0000977	RNA polymerase II regulatory region sequence-specific DNA binding	4.59E-04	25	18	1.99E-02	14	11
14	GO:0043565	sequence-specific DNA binding	1.03E-03	29	18	1.47E-02	18	12
15	GO:1990837	sequence-specific double-stranded DNA binding	1.24E-03	25	18	2.95E-02	14	11
16	GO:0008134	Transcription factor binding	1.06E-02	19	11	3.04E-02	12	6
17	GO:0140110	Transcription regulator activity	1.25E-04	36	20	1.77E-02	19	12
18	GO:0044212	Transcription regulatory region DNA binding	5.32E-04	26	19	1.73E-02	15	12
19	GO:0000976	Transcription regulatory region sequence-specific DNA binding	6.36E-04	25	18	2.27E-02	14	11
***Significant in Tg4-42 only***								
1	GO:0001216	DNA-binding transcription activator activity	3.00E-02	14	10	7.50E-02	9	7
2	GO:0001228	DNA-binding transcription activator activity, RNA polymerase II-specific	2.92E-02	14	10	7.85E-02	9	7
3	GO:0003700	DNA-binding transcription factor activity	1.06E-04	30	18	7.46E-02	14	11
4	GO:0140297	DNA-binding transcription factor binding	1.14E-02	13	8	7.67E-02	8	5
5	GO:0001217	DNA-binding transcription repressor activity	1.10E-02	11	8	1.00E+0	4	4
6	GO:0001227	DNA-binding transcription repressor activity, RNA polymerase II-specific	1.14E-02	11	8	1.00E+0	4	4
7	GO:0003690	Double-stranded DNA binding	1.30E-03	26	18	6.18E-02	14	11
8	GO:0043167	Ion binding	1.17E-02	77	30	8.46E-01	37	16
9	GO:0019904	Protein domain specific binding	4.26E-03	21	14	1.00E+0	6	4

*Benjamini–Hochberg False Discovery Rate (FDR).*

**TABLE 6:** GO annotation and pathway enrichment analysis of predicted miR targets on Biological Process.

			Tg4-42			WT		
				
	*Gene ontology*	*Label*	*FDR*	*No. genes*	*No. miRs*	*FDR*	*No. genes*	*No. miRs*
***Significant in Tg4-42 and WT***								
1	GO:0048856	Anatomical structure development	1.05E-05	85	32	2.28E-02	42	14
2	GO:0048646	Anatomical structure formation involved in morphogenesis	1.83E-02	21	11	2.61E-03	16	8
3	GO:0009653	Anatomical structure morphogenesis	8.34E-05	46	22	4.24E-05	30	12
4	GO:0048513	Animal organ development	1.22E-03	53	24	5.78E-03	31	12
5	GO:0007420	Brain development	1.59E-03	19	11	2.81E-03	13	6
6	GO:0000902	Cell morphogenesis	4.41E-02	17	11	3.64E-02	12	7
7	GO:0032989	Cellular component morphogenesis	3.30E-02	19	12	1.17E-02	14	8
8	GO:0016043	Cellular component organization	8.48E-05	80	24	5.23E-03	43	11
9	GO:0071840	Cellular component organization or biogenesis	1.67E-04	81	24	6.33E-03	44	11
10	GO:0007417	Central nervous system development	1.44E-02	20	12	3.08E-02	13	6
11	GO:0048598	Embryonic morphogenesis	2.44E-02	16	12	2.65E-03	13	8
12	GO:0007167	Enzyme linked receptor protein signaling pathway	2.72E-03	17	12	3.38E-03	12	6
13	GO:0060322	Head development	3.91E-03	19	11	5.83E-03	13	6
14	GO:0007275	Multicellular organism development	1.30E-04	77	29	4.27E-03	42	14
15	GO:0009890	Negative regulation of biosynthetic process	3.09E-05	37	23	4.01E-02	18	12
16	GO:2000113	Negative regulation of cellular macromolecule biosynthetic process	3.14E-05	35	22	4.08E-02	17	12
17	GO:0010629	Negative regulation of gene expression	1.15E-05	39	23	1.61E-02	20	13
18	GO:0010605	Negative regulation of macromolecule metabolic process	2.61E-05	50	23	4.26E-02	25	13
19	GO:0051172	Negative regulation of nitrogen compound metabolic process	2.58E-05	48	22	4.97E-02	23	12
20	GO:1903507	Negative regulation of nucleic acid-templated transcription	8.74E-05	31	20	2.82E-02	16	11
21	GO:0045934	Negative regulation of nucleobase-containing compound metabolic process	3.80E-05	35	20	1.84E-02	18	11
22	GO:1902679	Negative regulation of RNA biosynthetic process	8.73E-05	31	20	2.80E-02	16	11
23	GO:0051253	Negative regulation of RNA metabolic process	5.03E-05	33	20	2.14E-02	17	11
24	GO:0045892	Negative regulation of transcription, DNA-templated	8.63E-05	31	20	2.76E-02	16	11
25	GO:0007399	Nervous system development	1.52E-03	43	20	2.46E-02	24	10
26	GO:0048518	Positive regulation of biological process	9.98E-07	97	30	7.35E-05	52	15
27	GO:0009891	Positive regulation of biosynthetic process	4.06E-05	42	22	4.52E-05	27	12
28	GO:0031328	Positive regulation of cellular biosynthetic process	2.76E-05	42	22	3.37E-05	27	12
29	GO:0031325	Positive regulation of cellular metabolic process	9.67E-07	64	28	1.12E-05	38	14
30	GO:0048522	Positive regulation of cellular process	7.16E-07	90	28	1.78E-05	50	14
31	GO:0010628	Positive regulation of gene expression	9.37E-08	49	22	2.74E-05	28	12
32	GO:0010557	Positive regulation of macromolecule biosynthetic process	7.11E-06	42	22	4.21E-05	26	12
33	GO:0010604	Positive regulation of macromolecule metabolic process	1.33E-07	66	28	2.40E-05	37	14
34	GO:0009893	Positive regulation of metabolic process	7.36E-07	68	29	2.64E-05	38	14
35	GO:0051173	Positive regulation of nitrogen compound metabolic process	2.30E-06	61	28	1.51E-05	37	14
36	GO:1903508	Positive regulation of nucleic acid-templated transcription	3.00E-06	39	22	1.58E-05	25	12
37	GO:0045935	Positive regulation of nucleobase-containing compound metabolic process	1.21E-07	46	25	9.54E-06	28	13
38	GO:1902680	Positive regulation of RNA biosynthetic process	2.83E-06	39	22	1.37E-05	25	12
39	GO:0051254	Positive regulation of RNA metabolic process	2.66E-08	45	25	1.64E-05	27	13
40	GO:0045944	Positive regulation of transcription by RNA polymerase II	1.07E-03	28	16	4.64E-04	19	10
41	GO:0045893	Positive regulation of transcription, DNA-templated	7.21E-06	38	21	1.80E-05	25	12
42	GO:0022603	Regulation of anatomical structure morphogenesis	9.88E-04	27	16	3.42E-02	15	9
43	GO:0009889	Regulation of biosynthetic process	1.03E-08	73	28	6.46E-04	35	14
44	GO:0031326	Regulation of cellular biosynthetic process	9.92E-09	73	28	5.24E-04	35	14
45	GO:2000112	Regulation of cellular macromolecule biosynthetic process	1.02E-08	69	27	3.29E-04	34	14
46	GO:0031323	Regulation of cellular metabolic process	1.18E-08	96	30	3.35E-04	46	15
47	GO:0010468	regulation of gene expression	5.80E-09	78	27	6.35E-04	36	14
48	GO:0010556	Regulation of macromolecule biosynthetic process	9.78E-09	71	28	4.07E-04	34	14
49	GO:0060255	Regulation of macromolecule metabolic process	1.05E-08	96	30	1.33E-03	45	15
50	GO:0019222	regulation of metabolic process	1.26E-08	100	31	2.53E-03	46	15
51	GO:0051171	Regulation of nitrogen compound metabolic process	1.03E-08	92	30	3.23E-04	45	15
52	GO:1903506	Regulation of nucleic acid-templated transcription	3.18E-08	62	26	3.24E-04	31	13
53	GO:0019219	Regulation of nucleobase-containing compound metabolic process	2.72E-08	69	29	2.01E-04	35	14
54	GO:0080090	Regulation of primary metabolic process	9.49E-09	95	30	3.46E-04	45	15
55	GO:2001141	Regulation of RNA biosynthetic process	3.06E-08	62	26	3.16E-04	31	13
56	GO:0051252	Regulation of RNA metabolic process	9.26E-09	67	29	3.19E-04	33	14
57	GO:0006357	Regulation of transcription by RNA polymerase II	8.21E-07	46	23	5.09E-04	24	13
58	GO:0006355	Regulation of transcription, DNA-templated	6.53E-08	61	25	3.38E-04	31	13
59	GO:0048731	System development	3.77E-05	72	28	2.69E-03	39	13
60	GO:0009888	Tissue development	3.53E-02	30	17	3.96E-02	19	11
61	GO:0035239	Tube morphogenesis	2.66E-03	20	10	2.96E-02	12	7
***Significant in Tg4-42 only***								
1	GO:0009887	Animal organ morphogenesis	2.48E-02	22	15	2.10E-01	12	9
2	GO:0008306	Associative learning	4.10E-02	6	5	1.00E+00	2	1
3	GO:0007610	Behavior	1.71E-03	20	12	9.40E-01	7	5
4	GO:0065007	Biological regulation	7.24E-04	143	35	3.44E-01	67	16
5	GO:0048514	Blood vessel morphogenesis	2.02E-02	13	8	9.93E-01	5	4
6	GO:0048468	Cell development	1.12E-04	39	19	4.83E-01	16	9
7	GO:0030154	Cell differentiation	1.09E-03	61	26	4.39E-01	28	13
8	GO:0034330	Cell junction organization	5.87E-03	15	11	3.88E-01	7	4
9	GO:0048667	Cell morphogenesis involved in neuron differentiation	2.35E-02	13	9	1.14E-01	8	6
10	GO:0032990	Cell part morphogenesis	1.22E-02	15	11	8.20E-02	9	7
11	GO:0048858	Cell projection morphogenesis	7.70E-03	15	11	6.72E-02	9	7
12	GO:0048869	cellular developmental process	1.78E-03	61	26	4.59E-01	28	13
13	GO:0044260	Cellular macromolecule metabolic process	5.72E-04	63	24	5.02E-01	29	8
14	GO:0044237	Cellular metabolic process	4.38E-02	80	28	1.00E+00	33	9
15	GO:0009987	Cellular process	1.28E-02	155	37	1.00E+00	69	16
16	GO:0044267	Cellular protein metabolic process	2.83E-02	45	20	5.60E-01	22	8
17	GO:0006464	cellular protein modification process	8.05E-03	40	19	9.41E-02	22	8
18	GO:0070887	Cellular response to chemical stimulus	1.47E-03	44	19	2.56E-01	21	8
19	GO:0006974	Cellular response to DNA damage stimulus	2.24E-02	16	9	1.00E+00	3	3
20	GO:0071495	Cellular response to endogenous stimulus	3.29E-03	23	14	2.78E-01	11	6
21	GO:0071363	Cellular response to growth factor stimulus	1.19E-02	13	11	2.08E-01	7	5
22	GO:0071310	Cellular response to organic substance	1.70E-03	37	18	1.68E-01	18	8
23	GO:0033554	Cellular response to stress	2.60E-02	27	15	1.00E+00	8	6
24	GO:0050890	Cognition	8.16E-04	14	11	6.99E-01	5	4
25	GO:1904888	Cranial skeletal system development	4.16E-02	5	5	3.06E-01	3	3
26	GO:0007010	Cytoskeleton organization	1.23E-02	24	15	2.90E-01	12	5
27	GO:0048589	Developmental growth	2.96E-02	13	7	1.34E-01	8	6
28	GO:0032502	Developmental process	4.12E-05	87	32	6.05E-02	42	14
29	GO:0009790	Embryo development	3.48E-02	24	15	5.95E-02	15	9
30	GO:0060429	Epithelium development	2.60E-02	22	14	1.05E-01	13	10
31	GO:0007186	G protein-coupled receptor signaling pathway	1.82E-02	4	3	9.95E-01	3	2
32	GO:0035195	Gene silencing by miRNA	7.35E-03	5	5	6.48E-01	2	2
33	GO:0048699	Generation of neurons	1.40E-03	35	17	1.12E-01	18	10
34	GO:0040007	Growth	3.84E-02	13	7	1.51E-01	8	6
35	GO:0035556	Intracellular signal transduction	3.13E-03	30	15	8.90E-01	12	7
36	GO:0070059	Intrinsic apoptotic signaling pathway in response to endoplasmic reticulum stress	4.26E-02	4	4	6.05E-01	2	2
37	GO:0007612	Learning	6.44E-03	9	8	9.88E-01	3	2
38	GO:0007611	Learning or memory	2.69E-04	14	11	5.76E-01	5	4
39	GO:0007616	Long-term memory	6.81E-03	5	5	1.15E-01	3	3
40	GO:0043170	Macromolecule metabolic process	3.88E-04	76	28	9.76E-01	32	9
41	GO:0043412	Macromolecule modification	1.78E-02	41	19	1.84E-01	22	8
42	GO:0007613	Memory	2.35E-03	9	8	3.43E-01	4	4
43	GO:0035278	miRNA mediated inhibition of translation	2.69E-02	3	3	1.00E+00	1	1
44	GO:0050804	Modulation of chemical synaptic transmission	2.01E-04	19	12	8.98E-01	6	5
45	GO:0032501	Multicellular organismal process	1.82E-02	94	31	7.10E-01	45	14
46	GO:0061061	muscle structure development	2.24E-02	14	10	4.53E-01	7	5
47	GO:0048519	Negative regulation of biological process	7.27E-04	77	27	1.49E-01	38	13
48	GO:0009895	Negative regulation of catabolic process	4.44E-02	10	7	1.00E+00	3	3
49	GO:0010648	Negative regulation of cell communication	4.37E-02	26	12	1.00E+00	10	7
50	GO:0010721	Negative regulation of cell development	9.76E-03	13	8	1.00E+00	3	2
51	GO:0045596	Negative regulation of cell differentiation	1.89E-03	21	12	1.00E+00	6	5
52	GO:0034249	Negative regulation of cellular amide metabolic process	4.37E-02	7	7	1.00E+00	1	1
53	GO:0031327	Negative regulation of cellular biosynthetic process	1.75E-05	37	23	6.36E-02	17	12
54	GO:0031330	Negative regulation of cellular catabolic process	4.05E-02	9	6	1.00E+00	2	2
55	GO:0031324	Negative regulation of cellular metabolic process	3.16E-06	53	23	6.26E-02	24	12
56	GO:0048523	Negative regulation of cellular process	4.26E-04	72	25	1.06E-01	36	12
57	GO:0032269	Negative regulation of cellular protein metabolic process	4.24E-02	21	12	1.00E+00	8	6
58	GO:0051093	Negative regulation of developmental process	2.57E-03	25	14	7.96E-01	10	7
59	GO:1902532	Negative regulation of intracellular signal transduction	2.94E-02	14	8	8.35E-01	6	6
60	GO:0010558	Negative regulation of macromolecule biosynthetic process	4.95E-05	35	22	5.11E-02	17	12
61	GO:0051045	Negative regulation of membrane protein ectodomain proteolysis	1.03E-02	3	3	9.48E-02	2	2
62	GO:0009892	Negative regulation of metabolic process	3.02E-06	56	25	6.65E-02	26	13
63	GO:0051241	Negative regulation of multicellular organismal process	1.50E-02	27	16	5.08E-01	13	8
64	GO:0051961	Negative regulation of nervous system development	4.69E-02	11	7	1.00E+00	2	1
65	GO:0050768	Negative regulation of neurogenesis	3.01E-02	11	7	1.00E+00	2	1
66	GO:0043524	Negative regulation of neuron apoptotic process	2.88E-02	8	7	1.55E-01	5	5
67	GO:1901215	Negative regulation of neuron death	1.51E-02	10	9	1.29E-01	6	6
68	GO:0045665	Negative regulation of neuron differentiation	1.82E-02	10	7	1.00E+00	1	1
69	GO:2000635	Negative regulation of primary miRNA processing	3.82E-02	2	2	6.20E-01	1	1
70	GO:0048585	Negative regulation of response to stimulus	4.16E-02	29	13	1.00E+00	10	7
71	GO:0023057	Negative regulation of signaling	4.42E-02	26	12	1.00E+00	10	7
72	GO:0000122	Negative regulation of transcription by RNA polymerase II	3.74E-03	22	15	4.21E-01	10	8
73	GO:0017148	Negative regulation of translation	2.46E-02	7	7	1.00E+00	1	1
74	GO:0040033	negative regulation of translation, ncRNA-mediated	2.70E-02	3	3	1.00E+00	1	1
75	GO:0022008	Neurogenesis	8.64E-04	37	19	1.45E-01	18	10
76	GO:0048666	Neuron development	2.70E-03	22	13	1.69E-01	11	7
77	GO:0030182	Neuron differentiation	2.64E-03	25	15	5.69E-02	14	9
78	GO:0031175	neuron projection development	1.78E-03	20	13	1.46E-01	10	7
79	GO:0048812	Neuron projection morphogenesis	6.34E-03	15	11	6.01E-02	9	7
80	GO:0006807	Nitrogen compound metabolic process	4.11E-02	74	27	1.00E+00	32	9
81	GO:0006996	Organelle organization	3.94E-03	51	21	5.06E-02	28	9
82	GO:0016310	Phosphorylation	4.05E-02	21	14	1.00E+00	9	6
83	GO:0120039	Plasma membrane bounded cell projection morphogenesis	6.89E-03	15	11	6.34E-02	9	7
84	GO:0045597	Positive regulation of cell differentiation	1.50E-02	24	13	8.00E-02	14	8
85	GO:0030307	Positive regulation of cell growth	3.58E-02	8	6	5.61E-01	4	4
86	GO:0048639	Positive regulation of developmental growth	1.63E-03	11	8	6.76E-01	4	4
87	GO:0051094	Positive regulation of developmental process	1.57E-02	30	15	2.09E-01	16	9
88	GO:0045927	Positive regulation of growth	1.40E-03	13	9	1.00E+00	4	4
89	GO:1900273	positive regulation of long-term synaptic potentiation	1.60E-02	4	3	1.00E+00	1	1
90	GO:0061014	Positive regulation of mRNA catabolic process	1.56E-02	5	5	1.00E+00	1	1
91	GO:0048636	Positive regulation of muscle organ development	1.57E-02	6	4	1.00E+00	1	1
92	GO:1901863	Positive regulation of muscle tissue development	2.84E-03	7	5	1.00E+00	2	2
93	GO:0050769	Positive regulation of neurogenesis	3.52E-02	15	8	1.56E-01	9	5
94	GO:0045666	Positive regulation of neuron differentiation	1.66E-02	14	8	6.05E-02	9	5
95	GO:0010976	Positive regulation of neuron projection development	2.09E-02	12	8	6.07E-02	8	5
96	GO:1900153	Positive regulation of nuclear-transcribed mRNA catabolic process, deadenylation-dependent decay	6.12E-03	4	4	1.00E+00	1	1
97	GO:0060213	Positive regulation of nuclear-transcribed mRNA poly(A) tail shortening	1.98E-03	4	4	1.00E+00	1	1
98	GO:0045844	Positive regulation of striated muscle tissue development	1.56E-02	6	4	1.00E+00	1	1
99	GO:0050806	Positive regulation of synaptic transmission	8.45E-03	9	8	1.00E+00	3	3
100	GO:0009791	Post-embryonic development	2.00E-02	7	7	7.25E-01	3	3
101	GO:0016441	Posttranscriptional gene silencing	1.38E-02	5	5	7.50E-01	2	2
102	GO:0035194	Posttranscriptional gene silencing by RNA	1.29E-02	5	5	7.29E-01	2	2
103	GO:0019538	Protein metabolic process	4.44E-02	51	23	1.00E+00	22	8
104	GO:0036211	Protein modification process	7.99E-03	40	19	9.33E-02	22	8
105	GO:0006468	Protein phosphorylation	4.16E-02	17	11	4.21E-01	9	6
106	GO:0042981	Regulation of apoptotic process	2.00E-02	29	16	3.06E-01	15	9
107	GO:0050770	Regulation of axonogenesis	1.82E-02	9	6	6.59E-02	6	5
108	GO:0050789	regulation of biological process	1.53E-04	141	35	9.13E-02	67	16
109	GO:0065008	Regulation of biological quality	1.90E-05	69	23	1.68E-01	31	8
110	GO:0009894	Regulation of catabolic process	2.68E-03	22	15	3.68E-01	10	6
111	GO:0010646	Regulation of cell communication	1.14E-04	60	22	3.45E-01	26	12
112	GO:0060284	Regulation of cell development	2.47E-02	23	11	8.90E-01	10	5
113	GO:0045595	Regulation of cell differentiation	1.35E-03	38	17	6.79E-02	20	9
114	GO:0022604	Regulation of cell morphogenesis	4.39E-02	14	10	5.97E-01	7	5
115	GO:0042127	Regulation of cell population proliferation	2.30E-02	31	17	1.00E+00	10	7
116	GO:0034248	Regulation of cellular amide metabolic process	2.68E-02	12	9	1.00E+00	2	2
117	GO:0031329	Regulation of cellular catabolic process	1.96E-03	20	14	6.40E-01	8	5
118	GO:0051128	Regulation of cellular component organization	1.65E-03	46	23	1.50E-01	23	11
119	GO:0060341	Regulation of cellular localization	1.04E-03	23	11	6.31E-01	9	5
120	GO:0050794	Regulation of cellular process	8.35E-05	136	34	5.79E-02	65	15
121	GO:0032268	Regulation of cellular protein metabolic process	7.75E-03	44	23	4.38E-01	21	12
122	GO:0048638	Regulation of developmental growth	1.25E-02	13	9	8.77E-01	5	5
123	GO:0050793	Regulation of developmental process	5.53E-03	46	21	1.33E-01	24	10
124	GO:0040029	Regulation of gene expression, epigenetic	1.92E-02	9	7	1.00E+00	3	2
125	GO:0060968	Regulation of gene silencing	1.81E-02	6	6	1.00E+00	1	1
126	GO:0060964	Regulation of gene silencing by miRNA	7.93E-03	5	5	1.00E+00	1	1
127	GO:0060966	Regulation of gene silencing by RNA	1.02E-02	5	5	1.00E+00	1	1
128	GO:0040008	Regulation of growth	5.38E-03	19	12	1.00E+00	6	6
129	GO:0032879	Regulation of localization	5.41E-04	52	17	5.64E-02	27	8
130	GO:1900271	Regulation of long-term synaptic potentiation	2.85E-04	7	6	7.69E-01	2	2
131	GO:0042391	Regulation of membrane potential	2.93E-02	12	7	3.32E-01	7	3
132	GO:0065009	Regulation of molecular function	4.38E-02	41	20	3.15E-01	22	10
133	GO:2000026	Regulation of multicellular organismal development	6.79E-03	39	19	5.69E-02	22	10
134	GO:0051239	Regulation of multicellular organismal process	1.52E-02	51	23	8.43E-02	28	11
135	GO:0048634	Regulation of muscle organ development	1.89E-02	8	5	9.31E-01	3	3
136	GO:1901861	Regulation of muscle tissue development	4.60E-03	9	6	4.39E-01	4	4
137	GO:0051960	Regulation of nervous system development	3.96E-03	25	12	2.89E-01	12	6
138	GO:0050767	Regulation of neurogenesis	5.14E-03	23	11	5.93E-01	10	5
139	GO:0043523	Regulation of neuron apoptotic process	7.20E-03	11	9	1.56E-01	6	6
140	GO:1901214	Regulation of neuron death	7.69E-03	13	11	4.55E-01	6	6
141	GO:0045664	Regulation of neuron differentiation	2.62E-03	21	10	5.01E-01	9	5
142	GO:0010975	Regulation of neuron projection development	2.13E-02	16	9	4.40E-01	8	5
143	GO:0099601	Regulation of neurotransmitter receptor activity	3.27E-02	5	5	9.02E-01	2	2
144	GO:1900151	Regulation of nuclear-transcribed mRNA catabolic process, deadenylation-dependent decay	7.94E-03	4	4	1.00E+00	1	1
145	GO:0060211	Regulation of nuclear-transcribed mRNA poly(A) tail shortening	2.94E-03	4	4	1.00E+00	1	1
146	GO:0060147	Regulation of posttranscriptional gene silencing	1.03E-02	5	5	1.00E+00	1	1
147	GO:2000634	Regulation of primary miRNA processing	3.80E-02	2	2	6.19E-01	1	1
148	GO:0043067	Regulation of programmed cell death	2.26E-02	29	16	2.02E-01	16	9
149	GO:0032880	Regulation of protein localization	4.11E-02	22	13	2.95E-01	12	7
150	GO:0051246	Regulation of protein metabolic process	1.28E-02	45	23	5.12E-01	22	12
151	GO:0023051	Regulation of signaling	4.97E-05	61	22	3.54E-01	26	12
152	GO:0016202	Regulation of striated muscle tissue development	1.70E-02	8	5	9.25E-01	3	3
153	GO:0051963	Regulation of synapse assembly	1.86E-02	7	5	5.64E-02	5	3
154	GO:0050807	Regulation of synapse organization	2.61E-02	10	8	1.69E-01	6	4
155	GO:0050803	Regulation of synapse structure or activity	3.38E-02	10	8	1.98E-01	6	4
156	GO:0048167	Regulation of synaptic plasticity	2.92E-06	15	12	7.00E-01	4	4
157	GO:0045974	Regulation of translation, ncRNA-mediated	2.67E-02	3	3	1.00E+00	1	1
158	GO:0051049	Regulation of transport	1.26E-02	36	14	3.17E-01	18	6
159	GO:0099177	Regulation of *trans-*synaptic signaling	2.04E-04	19	12	8.99E-01	6	5
160	GO:0060627	Regulation of vesicle-mediated transport	4.23E-02	15	8	7.31E-01	7	2
161	GO:0042221	Response to chemical	1.26E-02	54	22	9.38E-01	24	8
162	GO:0009719	Response to endogenous stimulus	1.53E-03	27	15	1.87E-01	13	7
163	GO:0032354	Response to follicle-stimulating hormone	1.91E-02	3	3	1.45E-01	2	2
164	GO:0034698	Response to gonadotropin	4.15E-02	3	3	2.28E-01	2	2
165	GO:0070848	Response to growth factor	1.52E-02	13	11	2.37E-01	7	5
166	GO:0009725	Response to hormone	2.19E-02	16	11	4.43E-01	8	5
167	GO:0010033	Response to organic substance	4.12E-02	40	19	5.53E-01	20	8
168	GO:0050779	RNA destabilization	3.48E-02	4	4	1.00E+00	1	1
169	GO:0060021	Roof of mouth development	1.41E-04	9	8	5.24E-01	3	3
170	GO:0050808	Synapse organization	1.49E-02	11	9	1.00E+00	4	3
171	GO:0035295	Tube development	1.45E-03	24	12	6.01E-02	13	8
***Significant in WT only***								
1	GO:0016477	Cell migration	6.09E-01	14	7	1.15E-02	14	7
2	GO:0000904	Cell morphogenesis involved in differentiation	6.51E-02	14	9	1.90E-02	11	6
3	GO:0048870	Cell motility	4.92E-01	16	7	1.18E-02	15	7
4	GO:0051674	Localization of cell	4.93E-01	16	7	1.20E-02	15	7
5	GO:0040011	Locomotion	1.65E-01	21	10	8.95E-03	17	7
6	GO:0060485	Mesenchyme development	6.24E-02	8	6	1.82E-02	7	6
7	GO:0006928	Movement of cell or subcellular component	1.46E-01	24	11	1.84E-02	18	7
8	GO:0001843	Neural tube closure	4.55E-01	4	4	4.21E-02	5	5
9	GO:0051169	Nuclear transport	1.39E-01	7	4	1.54E-02	7	4
10	GO:0006913	Nucleocytoplasmic transport	1.39E-01	7	4	1.57E-02	7	4
11	GO:0050772	Positive regulation of axonogenesis	1.42E-01	5	4	4.71E-03	6	5
12	GO:0010770	Positive regulation of cell morphogenesis involved in differentiation	2.79E-01	6	4	1.12E-02	7	5
13	GO:0048729	Tissue morphogenesis	1.15E-01	14	10	3.68E-02	11	9
14	GO:0060606	Tube closure	4.63E-01	4	4	4.27E-02	5	5

*Benjamini–Hochberg False Discovery Rate (FDR).*

## Discussion

We performed NGS of the miRNome of the hippocampus of Tg4-42 mice, a model for sporadic AD. We assessed the pool of miRs before and after onset of AD-typical changes like gliosis, reduced glucose uptake into the brain, neuron loss and loss of reference memory (Bouter et al., 2013, 2019; Dietrich et al., 2018; Gerberding et al., 2019). The Tg4-42 mouse model is one of few mouse models developing neuron death in the CA1 region of the hippocampus (Bayer and Wirths, 2014), as such it might provide a powerful tool for preclinical drug testing and identification of the underlying molecular pathways driving AD pathology. The difference of diverse miR levels between 3 and 8 months of age in Tg4-42 elicits the AD-typical effects after onset of neuron loss and behavioral deficits. We cannot draw any conclusion on the difference in miR levels in aged mice, as the normal lifespan is at least 24 months.

At least 1% of the human genome encodes miR and every miR can regulate up to 200 mRNAs suggesting that dysregulation of miR expression could be associated with several human pathological conditions including central neurological disorders (Angelucci et al., 2019). In addition to NGS of the miRs, we have performed a search for the targets of identified miRs using state-of-the-art bioinformatics and addressed the question whether the affected processes are meaningful in the context of known AD-typical changes in the Tg4-42 mouse model.

### MiRs Identified in the Hippocampus of 8 Month Old Tg4-42 Mice

A total of 33 miRs were altered in the hippocampus between 3 and 8 month-old exclusively found in Tg4-42 mice. All but one was significantly decreased at the age of 8 months. Some of the miRs identified were already linked to AD pathology, while others could be associated to AD for the first time. In gray matter of the brain of patients with AD, down-regulation of a set of miRs (including several miR-15/107 genes and miR-29 paralogs) correlated strongly with the density of amyloid plaques. MiR-212 was found decreased in white matter, whereas miR-424 was upregulated in AD (Wang A. et al., 2011). Expression of miR-107 and BACE1 mRNA correlated with alterations in brain pathology in individuals with mild cognitive impairment (Wang et al., 2016). MiR-107 reversed the impairments of spatial memory and long-term potentiation caused by intraventricular injection of Aβ_1–42_ (Shu et al., 2018). MiR-125a may have a role in regulating the translation of PSD-95 mRNA. Impairments in the local synthesis of PSD-95, important for synaptic structure and function, may affect dendritic spine development and synaptic plasticity in fragile X syndrome (Ifrim et al., 2015). MiR-125b-1 induced tau hyperphosphorylation and cognitive deficits in AD (Banzhaf-Strathmann et al., 2014), may be involved in the regulation of inflammatory factors and oxidative stress by SphK1 (Jin et al., 2018). It has been found to be associated with other neurodegenerative diseases as well (Lardenoije et al., 2015; Salta and De Strooper, 2017b). TGF-β induced miR-100 and miR-125b (Ottaviani et al., 2019). MiR-100 and miR-125b coordinately suppress Wnt/b-catenin negative regulators, thereby increasing Wnt signaling (Lu et al., 2017). A pathogenic-positive feedback loop has been identified in which Aβ induced Dickkopf-1 expression activating non-canonical Wnt signaling, promoting synapse loss and enhancing Aβ production (Elliott et al., 2018). MiR-129-2 was reported to be down-regulated in spinal cord (Strickland et al., 2011). MiR-132 is involved in synaptic plasticity (Bicker et al., 2014) and may be associated with TDP-43 binding in amyotrophic lateral sclerosis (Freischmidt et al., 2013). Prion-infected hippocampal neurons elicited altered expression of miR-132 (Majer et al., 2012) and have been discussed to play an important role in inflammation control (Essandoh et al., 2016). MiR profiling in the human brain has revealed miR-132 as one of the most severely down-regulated miRs at the intermediate and late Braak stages of AD, as well as in other neurodegenerative disorders (Salta and De Strooper, 2017a). MiR-132 has been implicated in synaptic plasticity together with miR-134 and miR-138 (Bicker et al., 2014). Duplication of the miR-138-2 locus was observed exclusively in early onset AD cases and miR-138 overexpression *in vitro* induced Aβ production and tau phosphorylation (Boscher et al., 2019). Interestingly, levels of miR-132 and miR-138-2 were significantly decreased in aged Tg4-42 mice indicating that dendritic mRNA transport and local translation in the postsynaptic compartment play an important role in synaptic plasticity, learning and memory (Bicker et al., 2014) in this model system for AD. *In vitro* Aβ treatment increased the expression of miR-139 targets (Noh et al., 2014), and has been found to be associated with other neurodegenerative diseases (Lardenoije et al., 2015; Salta and De Strooper, 2017b). MiR-181c correlated with genome-wide DNA methylation changes of ncRNAs in patients with AD (Villela et al., 2016), and is involved in epigenetics of aging and neurodegeneration (Lardenoije et al., 2015). Overexpression of miR-185 inhibits autophagy and apoptosis of dopaminergic neurons by regulating the AMPK/mTOR signaling pathway in Parkinson’s disease (Bicker et al., 2014). The 22q11.2 deletion is a known genetic risk factor for schizophrenia and mouse models of 22q11.2DS have demonstrated down-regulation of miR-185 in key brain areas of affected individuals. This reduction was associated with dendritic and spine development deficits in hippocampal neurons (Freischmidt et al., 2013). Association studies in the 3′UTR of BACE1 and the miR-29 gene cluster did not identify an association with AD. A weak statistical interaction was observed between rs535860 (BACE1 3′UTR) and rs34772568 (near miR-29a). The authors concluded a major contribution of this miR (Bettens et al., 2009). MiR-30a has been found to be associated with Parkinson disease (Margis et al., 2011) as well as Huntington disease (Lardenoije et al., 2015; Salta and De Strooper, 2017b). MiR-338-3p depletion has been shown to be important for auditory thalamo-cortical signaling in 22q11DS mice, and may trigger the pathogenic mechanism of 22q11DS-related psychosis (Chun et al., 2017). MiR-345-5p may act as blood biomarker in multiple sclerosis (Freiesleben et al., 2016), and miR-345-3p attenuated apoptosis and inflammation caused by oxidized low-density lipoprotein by targeting TRAF6 via TAK1/p38/NF-kB signaling (Wei et al., 2020). An *in vitro* study indicated that miR-34a may inhibit Aβ clearance by targeting endophilin-3 including uptake and autophagy-mediated degradation (Sun et al., 2018). The rs1050283 SNP likely acts as a risk factor for sporadic AD. It is associated with a decreased expression of oxidized LDL receptor 1 mRNA in the absence of miR-369-3p de-regulation and may affect the binding of miR-369-3p to its 3′UTR consensus sequence (Bettens et al., 2009). MiR-381 showed a protective effect against inflammatory damage (Li et al., 2020). MiR-409 was reported in IL-17-induced inflammatory cytokine production in astrocytes by targeting the SOCS3/STAT3 signaling pathway in EAE mice (Liu et al., 2019). MiR-541 acts on neurite outgrowth and differentiation via nerve growth factor and synapsin I in neuronal precursor cells (Chun et al., 2017). MiR-674 demonstrated an association with Parkinson disease and Huntington (Lardenoije et al., 2015; Salta and De Strooper, 2017b). CSF from individuals with AD contained increased amounts of miR-let7b, and intrathecal injection of miR-let7b in wild-type mice induced neurodegeneration (Lehmann et al., 2012). Elevated levels of miR-let7b and miR-let7e were found in CSF of patients with AD and major depressive symptoms, but not in patients with fronto-temporal disorder (Derkow et al., 2018). MiR-let7d is a key regulator of bi-directionally transcribed genes mediating epigenetic silencing and nucleolar organization (Singh et al., 2018). For miRs miR-129-1, miR-154, miR-15b, miR-219-2, miR-298, miR-323, miR-411, miR-500, miR-7-2, miR-92b, miR-let7i this study is the first to describe deregulation in an AD mouse model. Interestingly, GO-Annotation analysis (“Cellular Components”) revealed that only miRs, which were exclusively deregulated in the Tg4-42 model showed an enrichment of targets in connection with the synapse. Such and similar annotations were completely absent among the targets of miRs that were deregulated in both aged mutant and WT mice. With respect to the “Molecular Function” and “Biological Process” annotation, this difference is even more pronounced.

As the Tg4-42 model shows reduced neurogenesis, synaptic hyperexcitability and develops neuron loss as well as behavioral deficits without plaque formation, the newly found deregulated miRs in this model could be involved in plaque-independent pathological pathways. Taken together these observations strongly support our conclusion that the identified miRs play a role in the etiology of AD and thus deserve further investigation as putative biomarkers or therapeutics.

### MiRs Identified in the Hippocampus of 8 Month Old Wildtype Mice

Eight miRs were exclusively altered in the hippocampus between 3 and 8 month-old in WT mice. TGF-β induced an lncRNA with its encoded miRs, miR-100 and miR-125b (Ottaviani et al., 2019). MiR-100 and miR-125b coordinately suppressed Wnt/β-catenin negative regulators thereby increased Wnt signaling (Lu et al., 2017). MiR-153 was over-expressed in CSF exosomes from patients with Parkinson disease (Serpente et al., 2011). There is evidence that miR-191 and miR-222 are co-regulated and are important for neurodevelopment (Li et al., 2020). MiR-1298, miR-34b, miR-423, miR-666 and miR-667 have not been previously reported to be association to AD, neurodegenerative disorders or brain function.

### MiRs Identified in the Hippocampus of 8 Month Old Tg4-42 and Wildtype Mice

25 miRs were differentially expressed between 3 and 8 month-old Tg4-42 as well as WT mice. All but three of them were significantly decreased at 8 months of age. MiR-127 has been reported to modulate macrophage polarization and therefore may have a crucial role in inflammation (Essandoh et al., 2016). MiR-128-1 up-regulation correlated with Aβ degradation in monocytes from patients with sporadic AD (Tiribuzi et al., 2014). MiR-130a was involved in inflammatory processes by targeting the transforming growth factor-beta1 and interleukin 18 genes (Zhao et al., 2014). MiR-132 expression levels were found to be associated with hippocampal sclerosis in a subgroup of AD patients (Tasaki et al., 2018). MiR-138-1 was transcriptionally up-regulated during myelination and downregulated upon nerve injury (Lin et al., 2018). miR-150-5p may act as a circulating biomarker for patients with the autoimmune neuromuscular disorder myasthenia gravis (Punga et al., 2015). A genetic variant located near the miR-204 gene was significantly associated with schizophrenia resulting in reduced expression of miR-204 in neuronal-like SH-SY5Y cells (Cammaerts et al., 2015). MiR-212 was down-regulated in white matter of patients with AD (Wang W.X. et al., 2011). MiR-222 was down-regulated in the AD mouse model APPswe/PSΔE9 (Wang et al., 2015). Lipopolysaccharide stimulation in mice led to increased expression of miR-221 and miR-222, thereby causing transcriptional silencing of a subset of inflammatory genes, which depend on chromatin remodeling. In patients with sepsis, increased expression of miR-221 and miR-222 correlated with immunoparalysis and increased organ damage (Seeley et al., 2018). Preliminary results indicated that aberrant levels of circulating miR-23a are recovered in fingolimod-treated multiple sclerosis patients representing a potential biomarker (Fenoglio et al., 2016). MiR-300 was involved in the inflammatory response in endothelial cells and enhanced autophagy by activation of the AMPK/mTOR signaling pathway (Li et al., 2018). MiR-323 suppressed neuron death via the transforming growth factor-β1/SMAD3 signaling pathway (Che et al., 2019). The expression of miR-30a was associated as a prediction serum marker in epilepsy (Lin et al., 2018). MiR-34c has been shown to have a physiological role in regulating the central stress response (Cammaerts et al., 2015). MiR-329 was upregulated in a mouse model for Rett syndrome, which is a complex neurological disorder that has been associated with mutations in the gene coding for Mecp2 (Urdinguio et al., 2010). MiR-382 inhibited cell proliferation and invasion of retinoblastoma by targeting BDNF-mediated PI3K/AKT signaling pathway (Song et al., 2017). MiR-877-3P regulated vascular endothelial cell autophagy and apoptosis under the high-glucose condition (Zhao et al., 2020). MiR-99a was found overexpressed in the brain of patients with Down syndrome (Bras et al., 2018). MiR-99b may have a role in spinal cord injury via the regulation of mTOR (Cao et al., 2017). Again, several miRs, namely, miR-128-2, miR-140, miR-23b, miR-30d, miR-3102, miR-328 and miR-434 could be linked to an AD mouse model for the first time in this study.

### Annotation Analysis of miR Targets

The analyses of the GO annotation of predicted miR targets in Tg4-42 and WT mice revealed diverse cellular component, molecular functions and biological processes similar to our previous study of the mRNome of the whole brain of Tg4-42 and 5XFAD mice (Bouter et al., 2014). The GO annotations in Tg4-42 hippocampus demonstrated that the most enriched pathways belong to synaptic signaling and transmission involved in memory processes, which was not found in WT mice. Hence, these pathways appear specific for the AD-typical mental decline and neurodegenerative events.

## Conclusion

We were able to validate the Tg4-42 as a valuable model for sporadic AD. We could confirm previously reported AD-associations of several miRs. Importantly, the untargeted small RNASeq approach also allowed us to link several additional miRs to a mouse model for AD. The identified miRs have a role in the age-dependent deficits in learning and memory as well as neuron loss in the hippocampus in Tg4-42 mice. The annotation of miR target genes supported these strong reductions in synaptic processes involved in learning and memory.

## Data Availability Statement

The datasets for this study have been uploaded to the European Nucleotide Archive (https://www.ebi.ac.uk/ena) with the accession identification number of the project PRJEB39314.

## Ethics Statement

The animal study was reviewed and approved by the Niedersächsisches Landesamt für Verbraucherschutz und Lebensmittelsicherheit, Röverskamp 5, 26203 Oldenburg, Germany and Landesamt für Gesundheit und Soziales LAGe So Darwinstr. 15, 10589 Berlin, Germany.

## Author Contributions

YB contributed to the experimental design and analyzed the data. TK and FR analyzed the data. AK and LJ contributed to the experimental design and were responsible for NGS-data generation and primary data analysis. TB wrote the manuscript, analyzed the data, and supervised the experimental design and the entire project. All authors read, reviewed, and approved the final manuscript.

## Conflict of Interest

The University Medicine Göttingen holds a patent on the Tg4-42 mouse model for Alzheimer’s disease.

## Footnotes

1 https://www.ebi.ac.uk/ena

## Supplementary Material

FIGURE S1 Heatmap of most variable genes for quality control. Individual sample after collapsing replicates with DESeq2. S1–20 represent individual sample labels.

TABLE S1 Analysis of hippocampal miR between young and aged Tg4-42 and wildtype mice. Plus log 2-fold change means higher levels at 8 months of age. Minus log 2-fold change means lower levels at 8 months of age. *P*-values are only shown if significant (<0.05). Abbreviations: 3M, 3 month old; 8M, 8 month old.
