# Supplementary figures and images for: miRNA Alterations Elicit Pathways Involved in Memory Decline and Synaptic Function in the Hippocampus of Aged Tg4-42 Mice
Source: Front Neurosci. 2020 Sep 10;14:580524. doi: 10.3389/fnins.2020.580524 (PMC7511553; doi:10.3389/fnins.2020.580524)

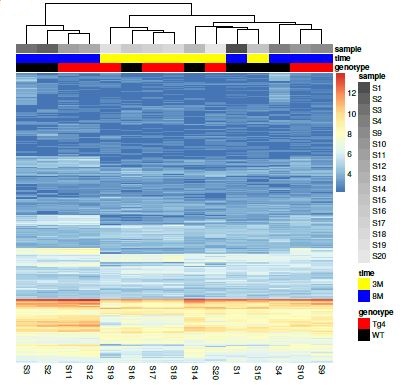

Supplement: FIGURE S1 — Heatmap of most variable genes for quality control. Individual sample after collapsing replicates with DESeq2. S1–20 represent individual sample labels. [file Image_1.JPEG]
