# Supplementary material for: miRNA Alterations Elicit Pathways Involved in Memory Decline and Synaptic Function in the Hippocampus of Aged Tg4-42 Mice
Source: Front Neurosci. 2020 Sep 10;14:580524. doi: 10.3389/fnins.2020.580524 (PMC7511553; doi:10.3389/fnins.2020.580524)
Supplement: TABLE S1 — Analysis of hippocampal miR between young and aged Tg4-42 and wildtype mice. Plus log 2-fold change means higher levels at 8 months of age. Minus log 2-fold change means lower levels at 8 months of age. P-values are only shown if significant (<0.05). Abbreviations: 3M, 3 month old; 8M, 8 month old. [file Table_1.DOCX]

# Supplementary Table 1. Analysis of hippocampal miR between young and aged Tg4-42 and wildtype mice. Plus log 2-fold change means higher levels at 8 months of age. Minus log 2-fold change means lower levels at 8 months of age. P-values are only shown if significant (<0.05). Abbreviations: 3M, 3 month old; 8M, 8 month old.

|  | **3M vs 8M Tg4-42** | | **3M vs 8M wildtype** | |
| --- | --- | --- | --- | --- |
| **miR** | **Log 2-fold change** | **p value** | **Log 2-fold change** | **p value** |
| miR-100 | -0.6 |  | -1.1 | 0.003 |
| miR-101b | 0.1 |  | -0.1 |  |
| miR-103-1 | -2.0 |  | -0.2 |  |
| miR-106b | -0.8 |  | -0.03 |  |
| miR-107 | -0.7 | 0.003 | -0.4 |  |
| miR-1193 | -1.7 |  | 0.7 |  |
| miR-1224 | 0.9 |  | -0.1 |  |
| miR-1247 | 2.0 |  | 0.1 |  |
| miR-1249 | -1.4 |  | -0.1 |  |
| miR-124a-3 | 0.2 |  | 0.02 |  |
| miR-125a | -1.2 | 0.0002 | -0.5 |  |
| miR-125b-1 | -1.4 | 0.0004 | -0.6 |  |
| miR-125b-2 | -0.7 |  | 0.0 |  |
| miR-126 | 0.03 |  | -0.5 |  |
| miR-1264 | -0.6 |  | -1.7 |  |
| miR-127 | -1.1 | 0.0003 | -0.7 | 0.03 |
| miR-128-1 | -1.7 | 1.8E-05 | -1.2 | 0.006 |
| miR-128-2 | -1.4 | 0.0001 | -1.5 | 0.0002 |
| miR-129-1 | -0.7 | 0.02 | -0.3 |  |
| miR-129-2 | -0.9 | 4.1E-05 | -0.4 |  |
| miR-1298 | -1.0 |  | -1.9 | 0.01 |
| miR-130a | -1.9 | 5.0E-05 | -1.3 | 0.01 |
| miR-130b | -0.5 |  | 0.5 |  |
| miR-132 | -2.5 | 3.6E-11 | -0.8 |  |
| miR-134 | -1.6 |  | -1.2 |  |
| miR-135a-2 | -1.8 |  | -0.7 |  |
| miR-135b | -0.4 |  | 0.7 |  |
| miR-136 | -0.4 |  | -0.3 |  |
| miR-137 | -0.3 |  | 0.3 |  |
| miR-138-1 | -0.9 | 0.006 | -0.8 | 0.04 |
| miR-138-2 | -1.0 | 0.0007 | -0.5 |  |
| miR-139 | -1.4 | 4.0E-05 | -0.7 |  |
| miR-140 | -0.9 | 0.001 | -1.0 | 0.002 |
| miR-142 | -0.3 |  | 0.3 |  |
| miR-143 | -0.2 |  | -0.4 |  |
| miR-144 | 0.7 |  | -0.04 |  |
| miR-145 | -1.3 |  | -2.1 |  |
| miR-146 | -0.7 |  | -0.2 |  |
| miR-148a | -1.0 |  | -1.0 |  |
| miR-148b | -1.4 |  | 0.7 |  |
| miR-149 | 0.04 |  | 0.5 |  |
| miR-150 | -1.8 | 1.4E-16 | -1.0 | 1.7E-05 |
| miR-152 | -0.2 |  | -0.9 |  |
| miR-153 | 0.9 |  | 1.2 | 0.04 |
| miR-154 | -0.9 | 0.02 | -0.2 |  |
| miR-15a | -3.0 |  | 0.6 |  |
| miR-15b | -1.2 | 0.04 | 1.6 | 0.05 |
| miR-16-1 | 0.1 |  | -1.2 |  |
| miR-16-2 | 0.2 |  | 0.1 |  |
| miR-17 | -0.5 |  | -0.1 |  |
| miR-18 | 0.6 |  | 0.2 |  |
| miR-181a-1 | -0.5 |  | 0.1 |  |
| miR-181a-2 | 0.3 |  | -0.4 |  |
| miR-181b-2 | -2.0 | 0.02 | -1.7 |  |
| miR-181c | -0.8 | 0.01 | -0.4 |  |
| miR-181d | -0.3 |  | 0.3 |  |
| miR-1839 | -0.4 |  | -0.4 |  |
| miR-185 | -0.6 | 0.02 | -0.2 |  |
| miR-186 | -0.7 |  | -0.1 |  |
| miR-187 | -1.1 |  | -0.6 |  |
| miR-188 | -0.5 |  | 0.7 |  |
| miR-190 | -1.5 |  | -0.6 |  |
| miR-191 | -0.5 |  | -0.7 | 0.03 |
| miR-1912 | 0.1 |  | -0.5 |  |
| miR-193 | -0.20 |  | 0.4 |  |
| miR-1930 | -0.4 |  | 0.3 |  |
| miR-193b | -1.2 |  | 0.8 |  |
| miR-194-2 | -2.0 |  | 0.2 |  |
| miR-195 | -0.1 |  | 0.04 |  |
| miR-1981 | -0.1 |  | -0.3 |  |
| miR-1982 | -0.8 |  | -1.0 |  |
| miR-1983 | -0.3 |  | -0.7 |  |
| miR-19a | 0.03 |  | -0.7 |  |
| miR-200a | 0.6 |  | 0.3 |  |
| miR-200b | 0.8 |  | -0.8 |  |
| miR-200c | 0.2 |  | 0.3 |  |
| miR-203 | -1.6 |  | 0.4 |  |
| miR-204 | -1.0 | 0.03 | -2.3 | 5.8E-06 |
| miR-205 | -0.2 |  | 0.2 |  |
| miR-20a | -1.5 |  | -1.5 |  |
| miR-210 | -0.5 |  | 0.8 |  |
| miR-212 | -1.7 | 7.0E-08 | -0.8 | 0.02 |
| miR-218-1 | -0.3 |  | -1.0 |  |
| miR-218-2 | -0.01 |  | -1.0 |  |
| miR-219-2 | -0.8 | 0.02 | -0.2 |  |
| miR-221 | -1.3 | 3.4E-07 | -1.0 | 0.0004 |
| miR-222 | -0.6 | 0.05 | -1.0 | 0.005 |
| miR-23a | -1.9 | 1.5E-10 | -2.6 | 7.5E-14 |
| miR-23b | -1.0 | 2.1E-06 | -0.7 | 0.003 |
| miR-24-1 | -0.3 |  | 0.5 |  |
| miR-24-2 | -0.7 |  | -0.7 |  |
| miR-25 | -1.2 |  | -1.3 |  |
| miR-26a-1 | -0.8 |  | 0.2 |  |
| miR-26b | -0.6 |  | -0.8 |  |
| miR-27a | -0.02 |  | -0.05 |  |
| miR-27b | -0.7 |  | -0.2 |  |
| miR-296 | -0.9 |  | -0.9 |  |
| miR-298 | -1.0 | 0.03 | -0.3 |  |
| miR-299 | -1.6 |  | 0.9 |  |
| miR-29a | -0.8 | 0.02 | 0.4 |  |
| miR-29b-1 | -2.0 |  | -0.9 |  |
| miR-300 | -1.0 | 9.8E-07 | -0.5 | 0.02 |
| miR-301 | -0.9 |  | 0.4 |  |
| miR-3059 | -0.7 |  | 0.2 |  |
| miR-3060 | -1.4 |  | 0.7 |  |
| miR-3068 | 1.1 |  | 0.2 |  |
| miR-3072 | 0.1 |  | 0.7 |  |
| miR-3076 | -1.0 |  | 0.4 |  |
| miR-3083 | -0.5 |  | 0.3 |  |
| miR-3085 | 0.3 |  | 0.04 |  |
| miR-3093 | 1.8 |  | 0.6 |  |
| miR-3096 | 1.4 |  | -0.5 |  |
| miR-3099 | -1.4 |  | 0.2 |  |
| miR-30a | -1.1 | 0.0006 | -0.7 | 0.03 |
| miR-30b | -0.7 |  | -0.4 |  |
| miR-30c-1 | -0.9 |  | -0.5 |  |
| miR-30d | -0.6 | 0.004 | -0.6 | 0.003 |
| miR-30e | -0.7 | 0.03 | -0.5 |  |
| miR-31 | -0.5 |  | 0.2 |  |
| miR-3102 | 1.2 | 0.0001 | 1.3 | 0.0002 |
| miR-320 | 0.1 |  | -0.04 |  |
| miR-323 | -1.1 | 0.005 | -0.6 |  |
| miR-324 | -0.04 |  | 0.2 |  |
| miR-326 | -0.01 |  | 0.6 |  |
| miR-328 | 0.7 | 0.02 | 1.8 | 5.9E-09 |
| miR-329 | -1.3 | 1.6E-05 | -1.0 | 0.003 |
| miR-33 | 1.0 |  | 0.9 |  |
| miR-330 | 0.3 |  | 0.2 |  |
| miR-331 | 0.2 |  | 0.4 |  |
| miR-335 | -0.5 |  | 1.0 |  |
| miR-337 | -1.2 |  | -0.5 |  |
| miR-338 | -0.6 | 0.05 | -0.1 |  |
| miR-339 | -0.3 |  | -0.1 |  |
| miR-340 | -0.8 |  | 0.3 |  |
| miR-341 | -0.4 |  | 0.3 |  |
| miR-344b | -0.2 |  | 0.01 |  |
| miR-344c | -1.4 |  | 0.2 |  |
| miR-345 | -0.8 | 0.006 | -0.6 |  |
| miR-346 | -0.4 |  | 0.5 |  |
| miR-3475 | -2.6 |  | -0.5 |  |
| miR-34a | -1.5 | 0.02 | -0.4 |  |
| miR-34b | -0.8 |  | -1.5 | 0.004 |
| miR-34c | -1.0 | 0.04 | -1.2 | 0.02 |
| miR-350 | 0.9 |  | -0.7 |  |
| miR-362 | -0.01 |  | 0.4 |  |
| miR-369 | -0.9 | 0.03 | -0.5 |  |
| miR-370 | 0.04 |  | -0.01 |  |
| miR-374 | 0.003 |  | -1.0 |  |
| miR-375 | -1.6 |  | -1.4 |  |
| miR-376a | 0.3 |  | 0.2 |  |
| miR-376b | -0.7 |  | 0.2 |  |
| miR-376c | -0.2 |  | -1.0 |  |
| miR-377 | -1.7 |  | -0.2 |  |
| miR-379 | -0.8 |  | -0.5 |  |
| miR-380 | -0.6 |  | -0.3 |  |
| miR-381 | -1.2 | 0.0004 | -0.2 |  |
| miR-382 | -1.1 | 0.005 | -1.1 | 0.02 |
| miR-383 | -1.1 |  | 0.1 |  |
| miR-384 | -0.4 |  | -0.01 |  |
| miR-409 | -1.1 | 0.003 | -0.6 |  |
| miR-411 | -1.1 | 0.006 | -0.3 |  |
| miR-423 | 0.3 |  | 1.0 | 0.003 |
| miR-425 | -0.6 |  | -0.5 |  |
| miR-429 | 1.3 |  | 1.0 |  |
| miR-431 | -0.5 |  | 0.1 |  |
| miR-434 | -1.2 | 0.0002 | -1.3 | 9.7E-05 |
| miR-448 | -0.5 |  | -1.7 |  |
| miR-451 | 0.5 |  | 0.4 |  |
| miR-455 | 0.3 |  | 0.6 |  |
| miR-483 | 0.5 |  | 1.6 |  |
| miR-484 | 0.2 |  | 0.4 |  |
| miR-485 | 0.04 |  | 0.4 |  |
| miR-487b | -0.6 |  | 0.01 |  |
| miR-488 | -0.3 |  | -1.9 |  |
| miR-490 | -0.4 |  | 0.3 |  |
| miR-491 | -0.7 |  | 0.7 |  |
| miR-494 | -1.0 |  | -1.6 |  |
| miR-495 | -0.1 |  | 0.5 |  |
| miR-496 | -1.5 |  | -0.2 |  |
| miR-497 | -0.7 |  | -1.0 |  |
| miR-500 | -0.8 | 0.02 | -0.7 |  |
| miR-501 | -0.3 |  | -0.8 |  |
| miR-504 | 0.5 |  | 1.3 |  |
| miR-505 | -0.4 |  | -0.4 |  |
| miR-5100 | -0.3 |  | 1.4 |  |
| miR-5109 | 0.9 |  | -0.02 |  |
| miR-532 | -0.3 |  | 0.3 |  |
| miR-539 | -0.7 |  | -1.5 |  |
| miR-540 | -0.1 |  | -0.7 |  |
| miR-541 | -0.9 | 0.04 | -0.8 |  |
| miR-543 | -0.9 |  | -0.5 |  |
| miR-551b | -0.01 |  | -0.1 |  |
| miR-574 | -0.3 |  | -0.4 |  |
| miR-582 | 0.03 |  | -0.4 |  |
| miR-592 | 0.5 |  | 1.3 |  |
| miR-598 | -0.7 |  | -0.1 |  |
| miR-665 | -0.2 |  | -0.02 |  |
| miR-666 | -0.2 |  | -1.4 | 0.006 |
| miR-667 | 0.6 |  | 1.6 | 2.2E-05 |
| miR-668 | -0.3 |  | 0.6 |  |
| miR-670 | -0.9 |  | -1.0 |  |
| miR-672 | -1.3 |  | -0.2 |  |
| miR-673 | -0.1 |  | 1.0 |  |
| miR-674 | -1.3 | 1.7E-06 | -0.6 |  |
| miR-676 | -1.1 |  | -1.0 |  |
| miR-677 | 2.2 |  | 0.9 |  |
| miR-700 | -0.2 |  | -0.1 |  |
| miR-708 | -0.7 |  | -0.4 |  |
| miR-7-1 | -0.3 |  | -0.5 |  |
| miR-7-2 | -1.3 | 0.02 | -1.1 |  |
| miR-744 | 0.2 |  | 0.5 |  |
| miR-758 | -0.5 |  | -2.0 |  |
| miR-760 | 0.1 |  | 0.8 |  |
| miR-764 | -0.4 |  | -0.2 |  |
| miR-7b | -0.5 |  | -0.5 |  |
| miR-872 | -1.0 |  | -1.2 |  |
| miR-873 | 0.3 |  | -0.3 |  |
| miR-874 | -0.7 |  | 0.1 |  |
| miR-877 | 0.8 | 0.02 | 1.5 | 0.0002 |
| miR-879 | -1.0 |  | -0.6 |  |
| miR-92b | 0.6 | 0.05 | 0.6 |  |
| miR-93 | -1.7 |  | -0.8 |  |
| miR-9-3 | 0.3 |  | 0.8 |  |
| miR-98 | -0.1 |  | 0.3 |  |
| miR-99a | -0.8 | 8.5E-04 | -1.3 | 4.3E-06 |
| miR-99b | -0.7 | 0.02 | -0.9 | 0.005 |
| miR-let7a-1 | -0.6 |  | 0.4 |  |
| miR-let7a-2 | -0.7 |  | 1.1 |  |
| miR-let7b | -1.2 | 0.0002 | -0.4 |  |
| miR-let7c-1 | 0.02 |  | -0.7 |  |
| miR-let7c-2 | 1.0 |  | 0.9 |  |
| miR-let7d | -0.7 | 0.001 | -0.1 |  |
| miR-let7e | -1.0 | 0.002 | -0.4 |  |
| miR-let7f-1 | -1.0 |  | -0.5 |  |
| miR-let7f-2 | -1.0 |  | -0.4 |  |
| miR-let7g | -0.3 |  | 0.02 |  |
| miR-let7i | -0.9 | 0.022 | -0.4 |  |
